# Smartphone and Instagram use, body dissatisfaction, and eating disorders: investigating the associations using self-report and tracked data

**DOI:** 10.1186/s40337-023-00865-1

**Published:** 2023-09-04

**Authors:** Dmitri Rozgonjuk, Johanna Ignell, Franziska Mech, Eva Rothermund, Harald Gündel, Christian Montag

**Affiliations:** 1 Department of Molecular Psychology, Institute of Psychology and Education, Ulm University, Helmholtzstraße 8/1, 89081 Ulm, Germany; 2 Institute of Mathematics and Statistics, University of Tartu, Tartu, Estonia; 3 Institute of Computer Science, University of Tartu, Tartu, Estonia; 4 Ulm University Medical Center, Department of Psychosomatic Medicine and Psychotherapy, Ulm University, Ulm, Germany

**Keywords:** Eating disorders, Body dissatisfaction, Instagram, Smartphone use, Social media use, Tracked data

## Abstract

**Background:**

Previous research has linked smartphone and Instagram use to higher body dissatisfaction (BD) as well as eating disorder (ED) symptomatology. However, these studies have typically been limited to using self-report measures for technology use which, as shown by scientific literature, might not be reliable. In the present work, we combine self-reported assessments as well as tracked smartphone and Instagram use.

**Methods:**

The effective sample comprised N = 119 women (34 with ED diagnosis history) who were queried about BD and ED symptomatology, and who provided the data about their smartphone and Instagram use duration for each day of the previous week.

**Results:**

The study results show that women with an ED diagnosis history scored higher on both BD as well as ED scales. Although women with an ED diagnosis history had higher smartphone screen time, there were no statistically significant differences in Instagram screen time. Tracked smartphone use duration was positively correlated with both BD and ED symptomatology, but the role of Instagram use needs to be further elucidated.

**Conclusions:**

The results of this study show that while BD and ED symptomatology are correlated with smartphone use, it may be that Instagram use is not the main contributor to that relationship.

## Abstract (plain language summary)

This study looked at how using smartphones and Instagram relates to feelings of dissatisfaction with one's body and symptoms of eating disorders. We compared women who had been diagnosed with an eating disorder in the past to those without such a history. We used information about the participants' smartphone and Instagram use from a tracking app, in addition to self-report surveys. The results showed that spending more time using smartphones was linked to higher levels of eating disorder symptoms and more body dissatisfaction. However, the connection between Instagram use and these psychological factors was not strong or consistent. There were no significant differences in Instagram use between women with and without an eating disorder diagnosis history. These findings suggest that smartphone use may be related to body dissatisfaction and eating disorder symptoms. At least according to the present data, Instagram use may not be the main driver of these issues. More research is needed to understand how to prevent and address these concerns, as well as to examine how different types of eating disorders relate to smartphone and social media use patterns.

## Background

Social media (SM) represents an environment where the pressure to demonstrate an ideal body characterized by physical activity, health, and attractiveness is especially emphasized. On SM, users construct an identity and present themselves in a specific manner [1]. Particularly, the SM platform Instagram is perceived as a communication environment of aesthetic visual content. Several users attempt to create an identity on Instagram that portrays a healthy lifestyle. To do this, they share health-related content, in which health and exercise become “life-stylized” ([1], p. 3). For instance, instead of merely sharing data from self-tracking technologies, it is considered more interesting to combine the self-tracking information with pictures of exercising in special locations. Creating such arranged presentations is labor-intensive and requires time. However, when others view these contents, they only see optimal, healthy, and attractive bodies.

The usage of SM, platforms on which users are constantly confronted with the idealized body, has been shown to be associated with heightened body image concerns [2]. As the number of SM users has increased over the past decade [3], it is interesting to investigate variables related to the SM usage, such as body dissatisfaction (BD) and eating disorder (ED) symptomatology. It is crucial to understand the aspects related to ED symptomatology, as EDs continue to be characterized by relatively high lifetime point prevalence (e.g., 8.4% and 2.2% for women and men, respectively; [4]), five times higher mortality rates compared to the general population [5], and high relapse rates of around 35% [6].

General media influence has been shown to strongly associate with internalization of thin body ideal, and this link is stronger than BD’s association with peer and parent influence [7]. As SM has the potential to combine general media influence with peer effects, it is not surprising that higher BD correlates with self-reported SM usage time [2, 8, 9]. Moreover, studying specific SM apps—which likely differ in the level they engage the user [10, 11]—could provide more insight into which type of media affects users’ body image the most. One such application is Instagram which is largely an image-based platform. Studies have linked self-reported Instagram use duration and frequency to higher BD [8, 9]. In a recent study, Fioravanti et al. [12] found that self-reported problematic Instagram use predicted more appearance comparison, which, in turn, predicted higher BD, potentially leading to increased ED symptomatology. BD is one of the leading risk factors for developing EDs [13–15].

The major methodological limitation of previous research on links between SM use, body image, and EDs is that the studies used self-reported (estimated) time and/or frequency of SM use. However, recent research in digital technology use has demonstrated the discrepancy between self-reports and tracked data [10, 16]. In the context of the study, „tracked data“ refers to data about participants' smartphone and Instagram usage obtained directly from their devices through built-in tracking applications [17]. These applications monitor and record the amount of time participants spend on their smartphones overall, as well as the specific duration of their Instagram use [18, 19].

On one hand, recent evidence has shown that the associations between self-reported and tracked digital technology use are weak [20]. On the other hand, psychological variables have been found to correlate more with self-reported but not so strongly (if at all) with tracked digital technology use [21, 22]. For instance, Rozgonjuk et al. [23] found that tracked Instagram use was not correlated with depression and anxiety, which contrasts findings based on self-reports [24, 25]. This suggests a significant gap in our understanding of the real impact of digital technology use on psychological variables (or vice versa). It raises the question of whether individuals who are more inclined to worry about their health are also more likely to perceive and report daily-life challenges related to their digital technology use. The distinction here is that this perception may not always align with actual, measured increases in their engagement with their smartphones or social media, as reflected in screen time data. This gap in our understanding also has implications for interpreting research findings. For instance, results from studies using self-reported data are often assumed to reflect actual behavior. For a more comprehensive understanding, it is beneficial to include both self-reported estimates and objectively tracked data (session logs) in future studies.

The aim of the present study is to explore the association between smartphone and Instagram use with BD and ED symptomatology in women, both with and without an ED diagnosis history. In order to address the methodological issues associated with relying on self-reports of smartphone and SM use, we also included tracked data (time spent on smartphone and Instagram). We pose the following hypotheses:

H1: *Smartphone use screen time is positively correlated with body dissatisfaction and eating disorder symptomatology.*

H2: *Instagram use screen time is positively correlated with body dissatisfaction and eating disorder symptomatology.*

Research has shown a consistent association between higher screen time, particularly on SM, and increased feelings of dissatisfaction with one's body and self-image [26, 27]. Furthermore, visually focused SM platforms, where the display of idealized bodies is prevalent, might lead to more frequent user comparisons [28]. Social comparison is a known critical risk factor for developing EDs [29]. Therefore, it is plausible to propose that both general smartphone screen time and, in particular, Instagram use may be associated with heightened ED symptoms.

Studies have suggested that Instagram may host various communities promoting unhealthy eating habits and idealized body images, which can potentially fuel ED symptomatology [26, 30, 31]. As mentioned earlier, Instagram, with its emphasis on presenting idealized, curated versions of life, can potentiate social comparison processes [28]. This is particularly relevant to individuals with EDs, who may engage in more upward social comparisons, contributing to heightened body dissatisfaction and ED symptoms [32, 33].

Moreover, individuals with ED history might present a unique relationship with Instagram use due to their past experiences. Those treated for EDs might have been advised to limit exposure to triggers like image-centric social media platforms, potentially reducing Instagram usage among this group to manage comparison effects.

On one hand, based on past findings [30, 32, 33], women with an ED history might use Instagram more. On the other hand, it could be that because these women might have worked with their ED already (i.e., have been in treatment), it could also be reflected in less Instagram use, as these women might have also reduced their Instagram use due to potential comparison effects (which may exacerbate EDs).

## Methods

### Procedure

German-speaking participants were invited to a web-based survey regarding their SM use. Participants were recruited primarily via online channels (e.g., SM, mailing lists, etc.); additionally, the opportunity to participate was also advertised in psychotherapy clinics in the area. The study participation inclusion criteria were being at least 18 years old, sufficient German-language proficiency to understand the survey items and owning and using an iOS or Android smartphone. The data were collected between March and May 2021, and an additional data collection was performed in December 2021 until February 2022.

The study was administered online via the web survey research platform SurveyCoder (https://ckannen.com) and took approximately 20–30 min to complete. Participation was voluntary and anonymous. Informed consent was given electronically prior to participation. All materials were presented in German.

The web survey included inventories regarding ED symptomatology, BD, problematic Instagram use (PIU), self-assessed Instagram use measuring how much time they typically spend on the app each day, and – if available – reports of tracked smartphone and Instagram use for the past seven days retrieved from a smartphone tracking application. Notably, the participants assessed their Instagram use times *before* inserting the respective times from the built-in smartphone tracking app to avoid the potential influence of seeing actual smartphone and Instagram usage on assessed times. More details on tracked data are given in the Sect. "Tracked smartphone and Instagram use".

The present study is a part of a larger project that was approved by the local institutional review board of Ulm University (protocol 512/20).

### Sample

Participants who (a) reported tracked Instagram use and (b) had reported these data for at least five days (out of seven) were included in the analyses (N = 205). However, within those participants, there were some smartphone and Instagram use duration values that were not plausible (i.e., the total Instagram use time was higher than the total smartphone usage time); therefore, these participants were also excluded, with N = 194 respondents remaining in the sample. Because tracked Instagram use duration is among the key variables in the focus of the present study, only those participants were included who did not mention limiting their daily Instagram use (i.e., the participants used a smartphone’s functionality to limit the use of Instagram; N = 141). Finally, we focused on the female sample,^1^ hence, the effective sample comprised N = 119 participants (ages 18–49, with M = 23.06, SD = 4.64).

Within the effective sample, most of the participants (N = 70; 58.82%) reported secondary education as their highest obtained education level, followed by Bachelor’s degree (N = 30; 25.21%). Other education levels were less represented. Sixty-four (53.78%) participants reported being in a relationship, 53 (44.54%) reported being single, and two (1.68%) participants reported being married. Twenty people (16.81%) had an iOS device, while most study participants (99; 83.19%) owned an Android smartphone.

Among the effective sample, 34 (28.57%) women reported either having a past or a present ED diagnosis. *Anorexia nervosa* was reported among 29 participants, *bulimia nervosa* in 16 participants, both binge eating and “other eating disorder” were reported by three participants, respectively. Importantly, diagnoses were not mutually exclusive. Sixteen (13.45%) were currently in an ED focused psychotherapy, 13 respondents (10.92%) had completed therapy in the past, while most of the study participants (N = 90; 75.63%) had no experience with ED related psychotherapy.

### Measures

As previously discussed, this study is a part of a larger project. We incorporated socio-demographic data, ED and body-image related items and inventories, as well as self-reported and tracked smartphone and Instagram use data.

#### Eating disorder diagnosis history and symptomatology

ED diagnosis history was asked with the following item: “Have you been diagnosed with an eating disorder in the past?” (response options: “yes” or “no”). In case the participant answered affirmatively, the participant was asked to select the EDs (*anorexia nervosa*, *bulimia nervosa*, binge-eating, or other; these options were not mutually exclusive).

ED psychopathology was assessed using the German version [37] of the Eating Disorder Examination-Questionnaire (EDE-Q; [38]. The EDE-Q is a self-report instrument, which comprises 28 items such as “On how many of the past 28 days have you had a strong desire to lose weight?”. Six items ask open-ended questions concerning diagnostically relevant information, e.g., number of episodes of self-induced vomiting, laxative misuse, or driven exercising. The rest of the items are answered on a seven-point Likert-scale indicating the frequencies and intensities of ED-specific symptoms in the preceding 28 days (0 = *feature was absent* to 6 = *feature was present every day* or *to an extreme degree*). The latter 22 items are used to form four subscales, namely Restraint (RS), Eating Concern (EC), Weight Concern (WC), and Shape Concern (SC). A global score can be calculated by averaging the subscale means. For the German version of the EDE-Q, Hilbert et al. [37] reported internal consistencies between α = 0.85 (WC) and α = 0.93 (SC) for the subscales and a Cronbach’s alpha of α = 0.97 for the global score. For the present study, the internal consistencies were as follows: α(RS) = 0.90, α(EC) = 0.90, α(WC) = 0.88, α(SC) = 0.96. Cronbach’s α for the global score was α = 0.97; the Spearman correlations between scale scores varied from r = 0.705 (between RS and EC) to r = 0.963 (SC and global score).

#### Body dissatisfaction

Body dissatisfaction was assessed with the German version [39] of the Body Shape Questionnaire (BSQ; [36]). The BSQ consists of 34 items such as “Have you thought that your thighs, hips or bottom are too large for the rest of you?”. Responses are given on a six-point Likert scale ranging from 1 (*never*) to 6 (*always*). Pook et al. [40] reported an internal consistency of α = 0.97 for a representative sample of German females. In the current study, Cronbach’s alpha for the BSQ was α = 0.98.

#### Self-assessed duration of smartphone and Instagram use

The participants were asked about their smartphone and SM use habits. Of interest to the present study, they were also asked to assess their daily smartphone and Instagram use in minutes.

#### Problematic Instagram use (PIU)

For the PIU, the Bergen Instagram Addiction Scale (BIAS; [41]), adapted from the Bergen Social Media Addiction Scale [42], was used. The BIAS is a six-item inventory that reflects the severity of PIU symptoms. The items show the agreement with given statements on a five-point scale (1 = *very rarely* to 5 = *very often*). The internal consistency within the effective sample for the scale was acceptable, α = 0.73.

#### Tracked smartphone and Instagram use

In the present study, to get objective data on smartphone and Instagram usage, the built-in smartphone applications *Screen Time* (iOS smartphones) and *Digital Wellbeing* (Android smartphones) were used. *Screen Time* and *Digital Wellbeing* are preinstalled smartphone apps, which allow users to keep track of their screen time behavior. Among other things, they provide a summary of smartphone usage times per day as well as individual usage times per day for each app that was used [18, 19]. In the present study, participants were asked whether one of those functions was installed and active on their phone. In case the app was actively running on the participant’s smartphone, they were asked to report the usage times (in minutes per day) of different (e.g., SM, calorie-tracking) apps relevant for the project.^2^ The participants were asked to report the data for seven full days before the day of survey. Previous research showed that collecting objective data for a minimum of five days is sufficient to gain a reflection of typical weekly smartphone usage [43].

We computed the average per day of smartphone as well as Instagram use minutes across the seven days. To investigate how the proportion of time spent on Instagram in relation to general smartphone use is associated with other variables, we also computed the proportion of Instagram use in relation to total smartphone use across seven days for each participant.

### Analysis

Statistical analysis was conducted in R software v4.3.0 [44]. The analyses were conducted both for the total sample as well as separately for women who reported being diagnosed or undiagnosed with an ED. There were no missing data in key variables of the study. To investigate the associations between BD, ED symptomatology, and smartphone and Instagram use, Spearman correlations were computed (p-values were adjusted with Holm’s method). For internal consistency statistics and correlation analysis, the *psych* package v2.3.3 [45] was used. Mann–Whitney U-tests (using the R’s *base* package) were used for comparing the differences in psychological variables and smartphone and Instagram use in samples of women with and without an ED diagnosis history.

## Results

### Descriptive statistics and correlations

The descriptive statistics and correlations are presented in Tables 1, 2, and 3 for the total sample as well as for the samples with and without an ED diagnosis history, respectively.

**Table 1:** Descriptive statistics and Spearman correlations for the total sample (N = 119)

	Descriptive statistics				Correlations					
Variable	*M*	*SD*	*Min*	*Max*	1	2	3	4	5	6
1. Smartphone use (OBJ)	266.09	149.97	49.71	1120.14	–					
2. Instagram use (OBJ)	59.51	36.36	5.43	183.14	.410***	–				
3. Instagram % (OBJ)	0.25	0.14	0.03	0.76	− .309*	.678***	–			
4. Instagram time (SR)	30.94	44.32	2	250	.271	.386***	.192	–		
5. PIU (SR)	13.99	4.46	6	30	.231	.295*	.153	.311*	–	
6. BSQ	88.68	40.97	34	204	.386***	.152	− .156	.202	.300*	–
7. EDE-Q: Restraining	2.88	1.84	1	7	.382***	.195	− .126	.091	.140	.742***
8. EDE-Q: Eating Concern	2.31	1.62	1	6.80	.280	.134	− .055	.136	.307*	.793***
9. EDE-Q: Weight Concern	3.23	1.83	1	7	.434***	.174	− .155	.225	.318*	.871***
10. EDE-Q: Shape Concern	3.57	1.89	1	7	.405***	.148	− .193	.178	.294*	.926***
11. EDE-Q: Global Score	3.00	1.68	1	6.70	.420***	.184	− .156	.171	.278	.904***

*Notes*. OBJ = tracked data (average minutes of screentime across seven days); SR = self-reported; PIU = problematic Instagram use; BSQ = Body Shape Questionnaire (body dissatisfaction scale); EDE-Q = (Eating Disorder Examination-Questionnaire; Min and Max = empirical minimum and maximum values, respectively. P-values are adjusted for multiple testing (with Holm’s method). * p < .05, ** p < .01, *** p < .001

**Table 2:** Descriptive statistics and Spearman correlations for the sample with an eating disorder diagnosis history (N = 34)

Variable	Descriptive statistics				Correlations					
	*M*	*SD*	*Min*	*Max*	1	2	3	4	5	**6**
1. Smartphone use (OBJ)	318.46	160.00	52.00	814.29	–					
2. Instagram use (OBJ)	68.79	40.24	9.71	183.14	.520	–				
3. Instagram % (OBJ)	0.23	0.11	0.06	0.52	− .174	.691***	–			
4. Instagram time (SR)	27.79	31.58	3	140	.514	.393	.056	–		
5. PIU (SR)	15.06	4.98	6	30	.422	.321	.009	.362	–	
6. BSQ	120.24	43.16	44	204	.214	.021	− .064	.371	.083	–
7. EDE-Q: Restraining	4.52	1.91	1	7	.243	− .030	− .140	.163	.143	.723***
8. EDE-Q: Eating Concern	3.84	1.72	1.20	6.80	.120	− .141	− .162	.231	.041	.673***
9. EDE-Q: Weight Concern	4.62	1.81	1.20	7	.276	.103	− .064	.301	.258	.811***
10. EDE-Q: Shape Concern	5.14	1.69	1.75	7	.260	.023	− .130	.412	.150	.869***
11. EDE-Q: Global Score	4.53	1.63	1.42	6.70	.223	− .029	− .128	.268	.161	.847***

OBJ = tracked data (average minutes of screentime across seven days); SR = self-reported; PIU = problematic Instagram use; BSQ = Body Shape Questionnaire (body dissatisfaction scale); EDE-Q = (Eating Disorder Examination-Questionnaire; Min and Max = empirical minimum and maximum values, respectively. P-values are adjusted for multiple testing (with Holm’s method). * p < .05, ** p < .01, *** p < .001

**Table 3:** Descriptive statistics and Spearman correlations for the sample without an eating disorder diagnosis history (N = 85)

Variable	Descriptive statistics				Correlations					
	*M*	*SD*	*Min*	*Max*	1	2	3	4	5	**6**
1. Smartphone use (OBJ)	245.14	141.36	49.71	1120.14	–					
2. Instagram use (OBJ)	55.79	34.23	5.43	169.71	.287	–				
3. Instagram % (OBJ)	0.26	0.15	0.03	0.76	− .354*	.717***	–			
4. Instagram time (SR)	32.20	48.59	2	250	.157	.363*	.221	–		
5. PIU (SR)	13.56	4.19	6	24	.115	.263	.193	.285	–	
6. BSQ	76.06	32.59	34	176	.324	.121	− .131	.183	.344*	–
7. EDE-Q: Restraining	2.22	1.34	1	7	.309	.168	− .086	.099	.071	.596***
8. EDE-Q: Eating Concern	1.70	1.10	1	6	.162	.131	.055	.172	.357*	.710***
9. EDE-Q: Weight Concern	2.68	1.52	1	6.60	.389**	.150	− .134	.263	.333	.819***
10. EDE-Q: Shape Concern	2.94	1.58	1	7	.345*	.118	− .179	.160	.311	.900***
11. EDE-Q: Global Score	2.38	1.25	1	6.44	.375*	.172	− .132	.181	.294	.874***

OBJ = tracked data (average minutes of screentime across seven days); SR = self-reported; PIU = problematic Instagram use; BSQ = Body Shape Questionnaire (body dissatisfaction scale); EDE-Q = (Eating Disorder Examination-Questionnaire; Min and Max = empirical minimum and maximum values, respectively. P-values are adjusted for multiple testing (with Holm’s method). * p < .05, ** p < .01, *** p < .001

Table 1 shows that BD and ED symptomatology are statistically significantly positively correlated with tracked smartphone use, yielding mostly medium effect sizes. The associations between BD and ED symptomatology yield high correlations. Interestingly, self-reported problematic Instagram use had a medium-effect positive correlation with BD, and the associations with ED symptoms were also mostly significant and with medium effects.

Tracked smartphone use was positively correlated with tracked Instagram use, but the proportion of Instagram use in relation to smartphone use was negatively correlated with smartphone use duration. In other words, the more time a person spends on a smartphone, the more the person spends time on Instagram—although the proportion of Instagram from time spent on smartphone decreases.

Finally, tracked Instagram use had small-to-medium effect correlations with both self-reported Instagram use duration as well as problematic Instagram use.

Table 2 displays these correlations for the sample that reported having an ED diagnosis history. Here, too, BD has high correlations with ED symptomatology variables. Although some of the ED symptomatology subscales (namely, weight and shape concern) yield medium-effect positive correlations with tracked smartphone use, these correlations are not statistically significant. However, within the sample without an ED diagnosis history, these associations are statistically significant—as was the association between the EDE-Q global score and smartphone use. Finally, problematic Instagram use was positively correlated with both BD as well as eating concern.

### Comparing women with and without an eating disorder diagnosis history

The differences in smartphone and Instagram use, BD, and ED symptomatology are displayed in Table 4.

**Table 4:** Differences between women with and without an eating disorder diagnosis history

Variable	Without diagnosis (N = 85)				With diagnosis (N = 34)				Differences test	
	*M*	*SD*	Median	*MAD*	*M*	*SD*	Median	*MAD*	*W*	*p*
1. Smartphone use (OBJ)	245.14	141.36	236	92.98	318.46	160.00	288.14	118.18	1925	**.005**
2. Instagram use (OBJ)	55.79	34.23	46.71	35.16	68.79	40.24	68.79	24.78	1713.5	.115
3. Instagram % (OBJ)	0.26	0.15	0.24	0.16	0.23	0.11	0.21	0.10	1284.5	.346
4. Instagram time (SR)	32.20	48.59	15	14.83	27.79	31.58	15	9.64	1483	.824
5. PIU (SR)	13.56	4.19	14	4.45	15.06	4.98	14.50	3.71	1685	.158
6. BSQ	76.06	32.59	69	31.13	120.24	43.16	123	55.60	2284	** < .001**
7. EDE-Q: Restraining	2.22	1.34	1.80	1.19	4.52	1.91	4.60	2.08	2391.5	** < .001**
8. EDE-Q: Eating Concern	1.70	1.10	1.20	0.30	3.84	1.72	4	2.22	2521.5	** < .001**
9. EDE-Q: Weight Concern	2.68	1.52	2.20	1.48	4.62	1.81	5.30	1.63	2285	** < .001**
10. EDE-Q: Shape Concern	2.94	1.58	2.50	1.30	5.14	1.69	5.94	1.39	2392	** < .001**
11. EDE-Q: Global Score	2.38	1.25	2.06	0.93	4.53	1.63	5.09	1.59	2400.5	** < .001**

OBJ = tracked data; SR = self-reported; PIU = problematic Instagram use; BSQ = Body Shape Questionnaire (body dissatisfaction scale); EDE-Q = (Eating Disorder Examination-Questionnaire; MAD = median average deviation. Statistically significant (p < .05) p-values are highlighted with **bold** font

First, the results clearly show that women with an ED diagnosis history score significantly higher on all ED symptomatology subscales, as well as BD. Second, it is noteworthy that although women with an ED diagnosis history, on average, have a significantly higher tracked smartphone use duration, these groups do not differ in terms of Instagram use variables. However, this interpretation of the results should be approached with caution since statistical significance is heavily affected by sample size.

## Discussion

The general aim of the present work was to provide insights into the potential links between smartphone use and, more specifically, Instagram use with BD and ED symptomatology. Additionally, women with an ED diagnosis history were compared in these variables with women without this psychopathology history. Although previous research has demonstrated the link between more Instagram use and higher ED symptomatology and BD [8, 9, 12], these works have relied on self-assessed Instagram use. The present work enriches these findings by including tracked smartphone and Instagram data for comparison. We formulated two hypotheses: firstly, we expected a positive correlation between BD, ED symptomatology, and smartphone use screen time (H1). Secondly, we anticipated a similar positive correlation between BD, ED symptomatology, and Instagram use screen time (H2). Additionally, we compared women with and without a history of ED diagnosis.

We found that smartphone use screen time is positively correlated with ED symptomatology. However, the relationship may be rather nuanced, as some ED symptoms (e.g., weight and shape concern) seem to have a stronger correlation with smartphone use than others (e.g., eating concern). Additionally, higher BD is associated with more smartphone screen time. However, in most cases, the effect sizes are not high. Hence, these results provide partial and nuanced support for the first hypothesis. It is also relevant to note that the more participants tended to use their smartphone, the more they used Instagram. However, the proportion of Instagram with regards to other apps and functions decreased.

Interestingly, Instagram use had small and/or non-significant correlations with both BD and ED symptomatology. These findings are somewhat surprising, since some of the recent works have demonstrated the generally positive link between Instagram use, BD, and ED symptomatology [8, 9, 12]. These findings may be explained in several ways. It could be that the association between Instagram use, ED symptomatology, and BD is not generally strong and robust. In other words, Instagram use may not necessarily be a good predictor for poorer body-image and EDs.

Alternatively, the operationalization of “Instagram use” could play a role—in the present work, we used the tracked Instagram screen time, self-reported Instagram use (in minutes), and a measure assessing problematic Instagram use (PIU). Of these approaches, only the PIU measure yielded statistically significant (positive) correlations with BD and ED symptomatology. The results of this study align with existing literature in the realm of smartphone use, which shows that while there may be positive small-effect correlations, self-reported problematic smartphone use often exhibits stronger connections with self-reported psychological variables than does objectively tracked smartphone use [10, 22]. This means that despite some modest correlations, tracked smartphone use often lacks robust or statistically significant associations with psychological outcomes. We do note that in some cases the analysis results were likely underpowered—nevertheless, statistical significance (or the lack of it) also came with respective effect sizes. And in the present study, the effect sizes were rather small—meaning that even if the effective sample was larger, the effects of links between tracked Instagram use and psychological variables would still be likely small to be considered relevant for practical application. This said, the larger association between PIU and BD/ED than with time spent on Instagram might also be traced back to the more clinical nature of the PIU scale.

Finally, we also explored if there were differences in Instagram use between women with and without an ED diagnosis history. Although women with an ED diagnosis history did have, on average, higher smartphone screen time, the differences in tracked Instagram use were not statistically significant. These findings seem to point to the possibility that the main difference between experiencing eating-related psychopathology does not reflect in Instagram use but rather in the total time spent on one’s smartphone. To our knowledge, previous research has not focused on this discrepancy before. Yet these findings are highly interesting because they suggest that something on the smartphone may engage women who have an ED diagnosis history more than women without an ED diagnosis history. Instagram use may play a role, but the influence of the use of several applications might add up.

The present study has several contributions. Firstly, it outlines the associations between BD, ED symptomatology, and smartphone and Instagram use. For instance, the results suggest that while smartphone use is more robustly linked to BD and ED symptomatology, this is not necessarily the case with Instagram use (at least in terms of objective Instagram use and effect sizes observed with ED/BD). Moreover, it could be observed that the proportion of time spent on Instagram from the total time spent on one’s smartphone does not correlate significantly with BD and EDs, suggesting that there might be other applications of more importance, driving the association between smartphone use, BD, and ED symptomatology. In addition to SM apps, calorie-tracking apps have received attention in relation to dietary patterns and body-image [46]. One could hypothesize that diet-tracking related activities on a smartphone could explain the association between smartphone use screen time, BD, and ED symptomatology.

A second important contribution of the present study is including tracked smartphone and Instagram use screentime to supplement self-reported Instagram use variables. Interestingly, there was a correlation between tracked smartphone use and both BD and ED symptomatology. Additionally, self-reported problematic Instagram use was associated with BD and ED symptomatology in the total sample as well as in women without an ED diagnosis history.

The main limitation of the present study is that the convenience sample could be larger; however, it should be viewed in the context of clinical as well digital technology use research. In comparison to regular online survey data, it may be difficult to gather tracked digital technology use—especially from a sample with an ED diagnosis history. To our knowledge, this is the first study that has achieved this, albeit on a rather small scale. The results of the present study, however, do suggest that several interesting patterns are present also with the smaller sample—providing rationale for further research.

Though our study provides valuable information about the relationships of interest in a female sample, it is important to note that the exclusion of men and non-binary individuals from our analyses can introduce biases and limit the generalizability of our findings. As gender can significantly influence the use and perception of social media [47], body dissatisfaction [48], and the prevalence of eating disorders [4], our results may not be fully applicable to populations beyond cisgender women. Future studies with a more diverse sample, including men and non-binary individuals, are needed to provide a more comprehensive understanding of these complex relationships.

It is also necessary to mention that the data were collected during the COVID-19 pandemic. Recent studies have shown that the pandemic could have effects on both psychological variables as well as digital technology use [49, 50]. Hence, it remains to be seen if the results of the present study can be replicated in post-pandemic settings.

While participants provided data on smartphone and Instagram use for several days via a built-in tracking app (either Digital Wellbeing on Android or Screen Time on iOS smartphones), these data were nevertheless self-reported (as the data needed to be typed into the survey environment). However, although some inaccuracies could be present (e.g., due to typing in the numbers retrieved from the phone screen) due to social desirability bias or reporting errors, there is little reason to believe that these data are of lower quality than data retrieved from designated tracking applications [51]. Although the questionnaire was implemented using a cross-sectional design, the data collected on smartphone and Instagram use over the previous week offers added value. Patterns of digital technology use, when considered over a more extended period, move beyond mere point-estimation, potentially yielding more reliable results. Nevertheless, future studies should go beyond relying on screentime measures—and should investigate the more specific activities conducted on one’s smartphone and Instagram app.

Looking ahead, if we acknowledge that smartphone use correlated with higher BD and elevated ED symptomatology, what are the possible interventions? One measure regards prevention: there are promising results in the improvement of body images via improving SM literacy [52, 53]. Although smartphone and SM use data may provide insights into a person’s psychological well-being (including experiencing ED symptoms), contemporary data-analytic approaches have also given promise for extracting meaningful knowledge from data-rich text data on SM [54–56]. Pairing both tracked smartphone and SM use data with semantic text analysis could not only describe the state of a person’s health but could also provide a necessary context for the person’s psychological characteristics.

Finally, it could be beneficial to distinguish between different EDs and explore their specific relations to digital technology use separately. As in SM research where different applications can drive higher or lower engagement and, hence, potential impact on daily-life [57], it could be that people with different EDs engage in using different (social) media and apps [58]. For instance, although tracking calories and fitness app use could be prevalent in all eating disorders [46], it might be hypothesized that people with *bulimia nervosa* or binge-eating disorder could be more drawn to platforms highlighting high-calorie foods, cooking recipes, or eating challenges, which could potentially trigger binge-eating episodes. Conversely, individuals with *anorexia nervosa* may be more likely to frequent pro-anorexia („pro-ana“) websites or sites that promote the „thin ideal“ [59]. Understanding these potential differential uses of digital technology could have significant implications for both the manifestation and the treatment of these disorders, possibly leading to more personalized interventions.

## Conclusions

The present study aimed to examine the relationships between smartphone and Instagram use with body dissatisfaction (BD) and eating disorder (ED) symptomatology, comparing women with and without a history of ED diagnosis. By incorporating tracked smartphone and Instagram data, the study provided insights beyond self-report measures. The findings showed positive correlations between smartphone use screen time and ED symptomatology, as well as higher BD. However, the associations between Instagram use and BD/ED symptomatology were small or non-significant, contrary to previous research. Differences in Instagram use between women with and without an ED diagnosis history were not statistically significant. The study sheds light on the nuanced relationships between smartphone use, Instagram, and psychological variables, suggesting that objectively-measured Instagram use is not a strong predictor of BD and EDs (i.e., at least in the present data; but also notice that *problematic* Instagram use shows larger associations). Further research is needed to explore prevention and intervention measures, as well as differentiate between specific eating disorders and their digital technology use patterns.

## Data Availability

The datasets used and/or analysed during the current study are available from the corresponding author upon scholarly request.

## Abbreviations

BD: Body Dissatisfaction
BIAS: Bergen Instagram Addiction Scale
BSQ: Body Shape Questionnaire
EC: Eating Concern (subscale of EDE-Q)
ED: Eating Disorder
EDE-Q: Eating Disorder Examination-Questionnaire
PIU: Problematic Instagram Use
RS: Restraining (subscale of EDE-Q)
SC: Shape Concern (subscale of EDE-Q)
SM: Social Media
WC: Weight Concern (subscale of EDE-Q)

## Footnotes

1 Given the disproportionately small number of men in the sample (22 out of 141), we decided to focus exclusively on the female participants (n = 119) for several reasons. Firstly, gender differences in body image perception and ED symptomatology are well-documented in the literature, with women typically experiencing higher rates of body dissatisfaction and EDs compared to men [34, 35]. Secondly, many studies cited in this work primarily or exclusively used female participants. By focusing on the female population, we were able to maintain a more homogeneous sample, thereby reducing potential confounding variables related to gender differences. Thirdly, the Body Shape Questionnaire (BSQ), one of the key measures used in this study, has been predominantly validated and utilized in female populations [36]. Since our study focused on body dissatisfaction in relation to Instagram use, using reliable and valid measures for our target population was crucial.

2 For Android smartphone users: Participants were provided with detailed instructions on how to retrieve relevant app usage data from the “Digital Wellbeing” feature in their device settings. The data of interest covered their app usage behavior over the past seven days, starting from the same day of the previous week up to the day before they retrieved the data. For iOS smartphone users: Similarly, participants using iOS devices were guided on how to extract the necessary information from the “Screen Time” section within their device settings. The timeframe of interest remained consistent with that of the Android users. We have not detailed the specifics of the data extraction process given the frequent updates and changes to both Android and iOS operating systems.
